# Pseudomyxoma Peritonei Originating from Transverse Colon Mucinous Adenocarcinoma: A Case Report and Literature Review

**DOI:** 10.1155/2020/5826214

**Published:** 2020-07-03

**Authors:** Yingbo Gong, Xin Wang, Zhi Zhu

**Affiliations:** Department of Surgical Oncology, Department of General Surgery, First Affiliated Hospital, China Medical University, Shenyang, China

## Abstract

**Background:**

Pseudomyxoma peritonei (PMP) is a rare neoplasm involving the peritoneum. Most PMPs are low-grade appendicular mucinous neoplasms (LAMNs). There have been no reports of PMP originating from a transverse colonic mucinous adenocarcinoma and causing metastatic mucinous adenocarcinoma. *Case Presentation*. We report a 46-year-old woman who presented with a right abdominal mass of more than 4-month duration. Transverse colonic mucinous adenocarcinoma, PMP, and ovarian metastatic mucinous adenocarcinoma were diagnosed. The patient's diet was normal, and she had no abdominal pain or bloating. The abdomen mass increased in the month before treatment. After chemotherapy, the transverse colon mass and ovarian giant cyst were resected and about 2000 mL of gelatinous tumor tissue was removed. Postoperative histology confirmed PMP from the transverse colonic mucinous adenocarcinoma, ovarian metastatic mucinous adenocarcinoma, and mesocolon metastatic cancer. Multiple lung metastases appeared 8 months after surgery. The patient died 29 months after surgery because of an inability to eat and poor nutrition. A systematic literature review of the management and outcome of all known similar cases is also presented.

**Conclusions:**

This is the first report of PMP originating from a transverse colonic mucinous adenocarcinoma. It was diagnosed during resective surgery, involved ovarian metastasis, and survival was short. We did an extensive literature review in order to describe the clinical characteristics, histopathological findings, genetic profile, and potential treatments of PMP caused by nonappendiceal mucinous adenocarcinoma.

## 1. Background

Pseudomyxoma peritonei (PMP) is a rare neoplasm that involves the peritoneum and has an estimated incidence of one to two cases per million per year [1]. It is characterized by the production of large amounts of mucinous ascites that can fill the peritoneal cavity [2]. Early-stage patients may be asymptomatic or have nonspecific symptoms including abdominal pain, bloating, and ascites. Accumulation of mucinous ascites following lesion rupture, release, and dissemination of tumor cells into the abdominal cavity may cause partial or complete bowel obstruction [3]. The lack of specific symptoms can delay diagnosis until relatively late in the clinical course. The primary tumor is most often an appendiceal mucinous tumor, or low-grade appendicular mucinous neoplasm (LAMN), but PMPs can derive from ovarian, gastric, or colorectal tumors [4, 5]. To the best of our knowledge, no cases of PMP induced by transverse colonic mucinous adenocarcinoma with ovarian metastasis have been reported. We present the first case of this rare entity originating from a transverse colonic mucinous adenocarcinoma and causing metastatic mucinous adenocarcinoma. A systematic literature review of the management and outcome of all known similar cases is also presented.

## 2. Case Presentation

A 46-year-old woman presented with a right abdominal mass of more than 4-month duration. She had a normal diet, no abdominal pain or bloating, and could defecate, but the stools had no formed elements. Weight loss was not apparent. The patient had no relevant medical history, denied tobacco, alcohol, or drug use, and there was no family history of cancer. Symptomatic treatment did not result in improvement, and the abdominal mass increased significantly in the month before additional treatment was started. The patient had a doughy abdomen with tenderness and a palpable fist-sized mass in the right upper quadrant without rebound tenderness or muscle tension. Borborygmus was normal, the Douglas space was full, no nodules were involved, and no systemic lymph nodes were swollen. Proliferative masses near the splenic flexure of the colon prevented the completion of a colonoscopy. The pathological diagnosis was high-grade intraepithelial neoplasm.

**Figure 1:**
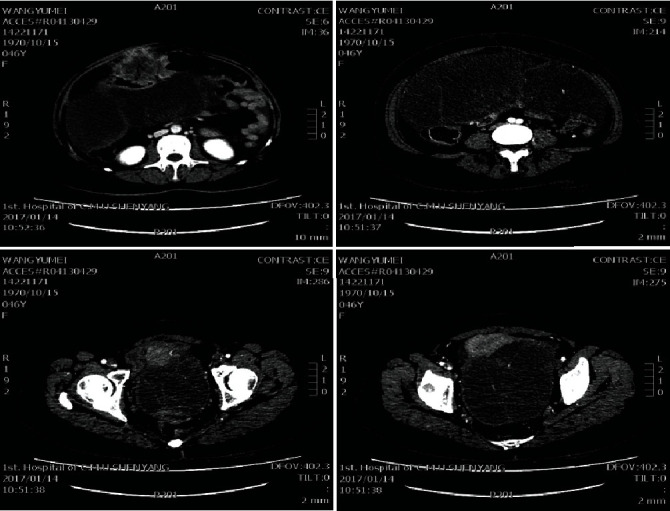
Abdominal enhanced CT shows irregular thickening of the wall of the hepatic flexure of colon with narrowing of the lumen and uneven enhancement.

A total abdominal enhanced computed tomography (CT) scan on January 12, 2016, showed irregular thickening of the wall of the hepatic flexure of the colon with narrowing of the lumen. Enhancement was uneven, the anterior abdominal wall was pushed forward, and the fat gap around it was blurred. The proximal ascending colon was slightly dilated, and a density shadow indicated that fluid surrounded the liver and spleen. Multiple nodules and mucoid densities were seen in the peritoneum and abdominal cavity; the septum was enhanced; mucoid lesions were not seen. There were clear enhancement and unclear demarcation of uterine and adjacent structures, and unclear imaging of uterine structures (Figure 1). The white blood cell count (10.16 × 10^9^/L) and neutrophil percentage (80%) were high; hemoglobin was low (97 g/L). C-reactive protein (CRP, 134 mg/L), C-reactive protein (CRP, 41 ng/mL), carbohydrate antigen 19-9 (CA19-9, 96 U/mL), and carbohydrate antigen 125 (CA125, 240 U/mL) were high.

**Figure 2:**
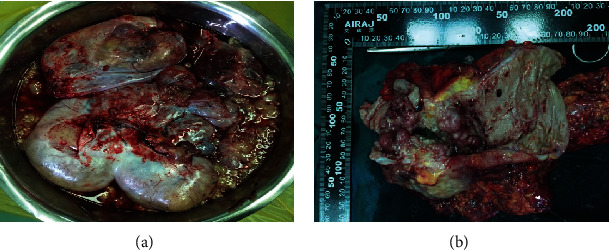
(a) Abundant mucous gelatinous tissue and ascites are seen in the abdominal cavity along with bilateral giant ovarian cysts of about 25 × 15 × 15 cm and (b) at 8 × 6 × 4 cm mass in the transverse colon that infiltrated and adhered to the abdominal wall.

**Figure 3:**
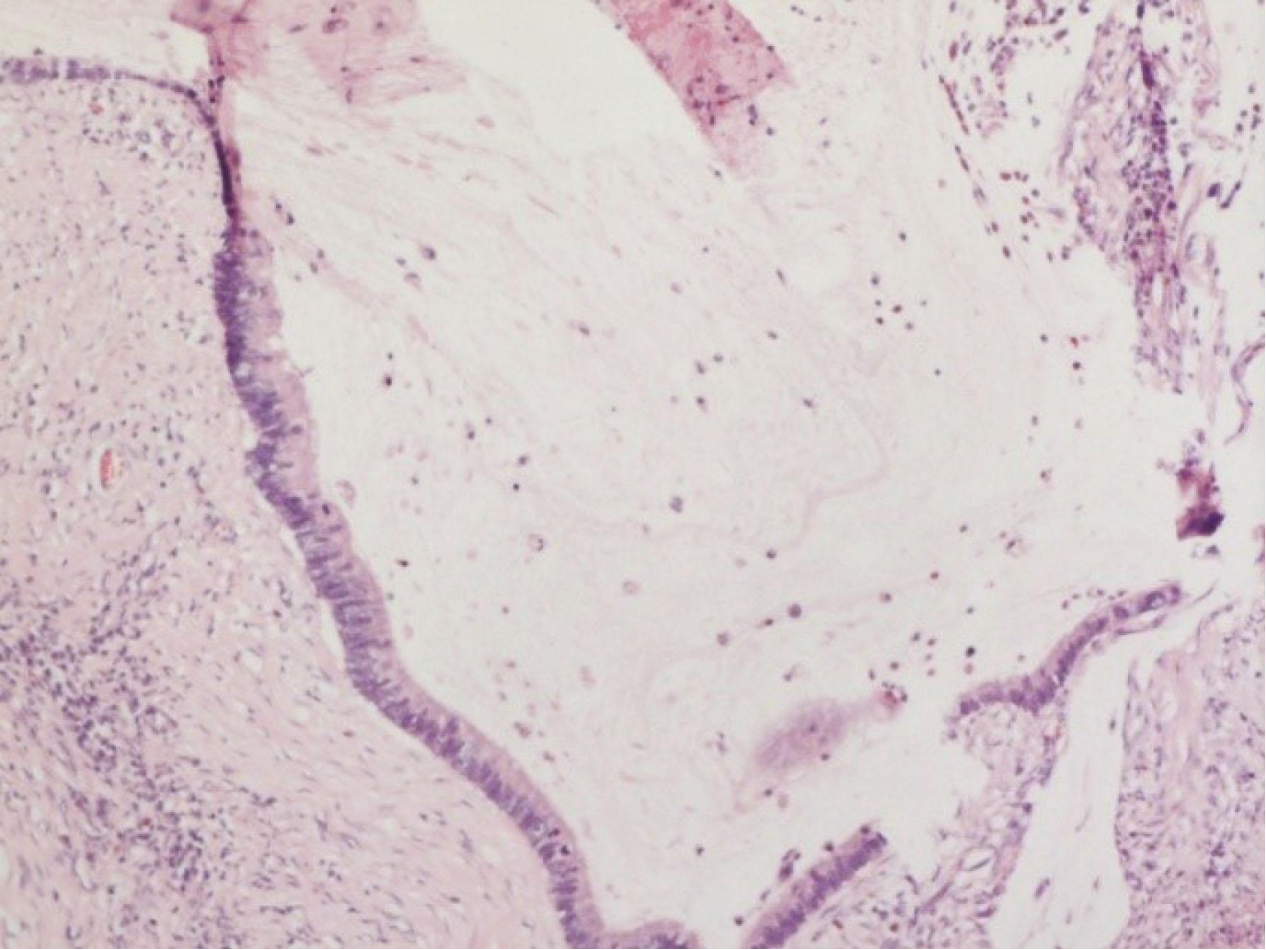
Postoperative gross pathology of a transverse colonic mucinous adenocarcinoma after cytoreductive surgery. Immunohistochemical staining of the tumor tissue was CK7(-), CK20(+), CA125(-), CA19-9(+), CDX-2(+), cadherin 17(+), P53(+++), PAX8(-), SATB2(+), beta-catenin(+), and 60% Ki-67(+).

Abdominal enlargement continued following admission to the department of medical oncology, and chemotherapy was difficult to administer. Cytoreductive surgery with follow-up treatment was recommended by a multidisciplinary team of medical and surgical oncology, gynecology, imaging, and radiotherapy specialists considering ovarian metastasis of the transverse colon tumor and extensive abdominal and pelvic implantation of the myxoma. Surgery was performed on January 20, 2017. An 8 × 6 × 4 cm transverse colon mass that had infiltrated and adhered to the abdominal wall, about 2000 mL of mucous gelatinous tumor tissue, ascites, and both ovarian cysts, were completely removed (Figure 2). Hyperthermic intraperitoneal chemotherapy (HIPEC) was not performed during surgery because of a lack of disagreement among the patient's family members. Postoperative pathology confirmed a pT4bNxM1b mucinous adenocarcinoma of the transverse colon with metastasis to the serosa of both ovaries and the mesocolon. Immunohistochemical staining found that the primary tumor was CK7(–), CK20(+), CA125(–), CA19-9(+), CDX-2(+), cadherin17(+), P53(+++), PAX8(–), SATB2(+), beta-catenin(+), and 60% Ki-67(+) (Figure 3). Genetic testing revealed that the patient carried mutations in codon 12 in exon 2 of the KRAS gene and a V600E BRAF mutation, but no NRAS mutation. Unfortunately, the patient did not consent to treatment with a targeted drug because of a lack of insurance coverage.

A follow-up evaluation in September 2017 after surgery and 1 month after six cycles of capecitabine plus oxaliplatin (XELOX) chemotherapy found multiple lung metastases. The patient was switched to an irinotecan, raltitrexed, and Avastin regimen, but treatment was stopped in February 2019 because the patient could no longer tolerate chemotherapy. The patient died in July 2019, 29 months after diagnosis, because of malnutrition and inability to eat.

## 3. Discussion

PMP is a rare disease involving the peritoneum, and it usually presents with a “jelly abdomen,” the presence of abdominal mucin with dispersed cancer cells. The first case was described by Rokitansky in 1842 [6]. It was found to be associated with ovarian cancer by Werth in 1884 and with appendiceal cystic tumors by Frankel in 1901 [7, 8]. PMP is caused by cells that are released into the abdomen from a primary adenoma and produce mucinous or gelatinous ascites [9]. The primary lesion can originate from almost any abdominal organ, but most often, it is an appendiceal mucinous tumor, or LAMN [10–13]. Reports of PMP deriving from rectal cancer or sigmoid colon cancer are very rare [14, 15] (Table 1).

Ronnett et al. described three types of PMP: disseminated peritoneal adenomucinosis (DPAM), which tends to form benign lesions; peritoneal mucinous carcinomas (PMCA), which often form malignant tumors; and an intermediate type that exhibits characteristics of both DPAM and PMCA (PMCA-I/D) [16]. DPAM lesions have abundant extracellular mucin and few single or focally proliferative mucinous epithelial cells with low-grade cytologic atypia and low mitotic activity. PMCA lesions are richer in mucinous epithelial cells with a tissue structure and cytological feature characteristic of cancer. DPAM and PMCA may be found with or without an associated primary mucinous adenocarcinoma [17]. The prognosis of PMCA and PMCA-I/D is significantly worse than that of DPAM. Ronnett et al. reported 5- and 10-year survival rates of 14% and 3% for PMCA-I/D and 75% and 68% for DPAM. In a group of 2298 PMP patients treated with cytoreductive surgery (CRS)+HIPEC, the 5- and 10-year survival rates were 59% and 49% for PMCA, 78% and 63% for PMCA-I/D, and 81% and 70% for DPAM [18]. Existing evidence shows that survival is associated with the nature of the primary tumors and the histology of the peritoneal tumors. The current pathological classification of PMPs thus helps physicians to predict patient prognosis [16]. The studies by Ronnett et al. and Chua et al. included only PMAC patients with appendiceal adenocarcinomas. The survival rate of PMAC patients with nonappendiceal mucinous adenocarcinomas is not clear. The prognosis of both reported cases of PMP caused by nonappendiceal mucinous adenocarcinomas, one in a patient with rectal cancer and the other with sigmoid colon cancer, was poor [14, 15]. Our case is the first report of a PMP caused by transverse colon cancer. It was a PMCA, and the prognosis was poor, with a survival of only 29 months. Additional experience with PMP caused by nonappendiceal mucinous adenocarcinomas is needed to determine the prognosis.

CEA, CA19-9, and CA125 are useful for the diagnosis of PMP, indicating the severity of the disease and predicting prognosis. The survival of CEA-, CA19-9-, and CA125-negative patients after surgery was found to be 2.6 times longer than that of patients who were positive for all three markers [19–21]. This patient and the previously reported patient with sigmoid colon cancer were positive for all three markers. The patient with rectal cancer was positive for CEA [14, 15]. All three patients had a poor prognosis.

A recent study described molecular profiles of PMP that included frequent mutations in KRAS, NRAS, and BRAF fusion products, deregulation of the TP53 or PI3K-AKT pathways in PMCAs but not DPAMs, decreased expression of E-cadherin, and amplification of MCL1 and JUN [22]. However, the precise molecular mechanisms underlying the development and progression of PMPs and the genetic factors associated with the response to treatment remain to be resolved.

The BRAF gene is mutated in ∼7% of human cancers, including colorectal, melanoma, papillary thyroid, and non-small-cell lung cancer with variable frequency and allelic distribution. The valine substitution at residue 600 (V600) accounts for >90% of the BRAF mutations. Activating BRAF mutations and fusion products constitutively activates the MAPK/ERK pathway (also known as the RAS-RAF-MEK-ERK pathway) that communicates receptor signaling to the DNA in the nucleus of the cell [23]. Drugs that target those mutations might have been effective in this patient. Cetuximab and panitumumab, both of which are monoclonal antibodies against the epidermal growth factor receptor (EGFR), have been proven effective for patients with RAS wild-type metastatic colorectal cancer (mCRC) in clinical trials, with higher response rates and longer progression-free survival than chemotherapy. However, only a small percentage of mCRC patients are sensitive to anti-EGFR therapy, and even those who initially respond to the therapy eventually develop resistance to it [24].

**Figure 4:**
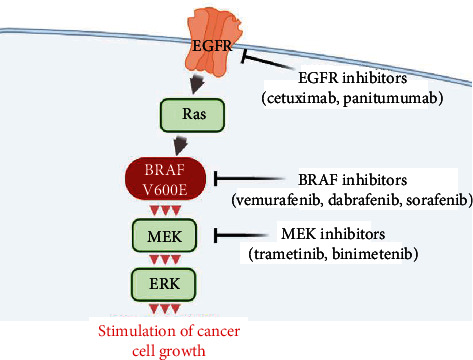
Representative drugs for MAPK/ERK pathway and BRAF v600E mutation for potential therapy of PMP from mCRC.

Evidence from clinical trials shows that patients with BRAF-positive phenotypes can benefit from treatment with tyrosine kinase inhibitors (TKIs). Two selective BRAF inhibitors, vemurafenib and dabrafenib, have been approved by the United States Food and Drug Administration for the treatment of BRAF V600-positive cancer. Two phase III studies reported objective response rates of ~50% and a median progression-free survival of ~5.3 months for vemurafenib and dabrafenib in patients with BRAF V600-positive melanoma [25, 26]. Trametinib is a mitogen-activated protein kinase (MAP2K or MEK) inhibitor that was shown to be effective as a single agent in V600 BRAF-mutated cells in NSCLC [27]. These TKIs have not yet been evaluated in mCRC clinical trials. Evidence of effectiveness against PMP from colorectal cancer (CRC) is limited, but a treatment strategy based on that used for melanoma and NSCLC is proposed in Figure 4.

For patients with an aggressive form of mCRC, a treatment regimen that includes a combination of targeted cancer drugs can be beneficial. Evidence from the BEACON CRC trial, a phase III study of a three-drug regimen that combined encorafenib with cetuximab plus binimetinib to treat an aggressive form of mCRC with BRAF V600E mutation improved survival without increasing the risk of serious side effects [28]. Those results illustrate the importance of genetic analysis to direct the choice of therapy for mCRC patients. The 2018 National Comprehensive Cancer Network (NCCN) guidelines for the treatment of PMP patients state that the current evidence shows CRS but not hyperthermic intraoperative peritoneal chemotherapy (HIPC) is correlated with an improvement in overall survival [29]. The available results may not be conclusive and a standard treatment is not available, but recent studies found that combining HIPEC and CRS resulted in the best outcomes in PMP patients [30–32]. HIPEC was not performed in this patient during surgery because of a lack of agreement among family members, and that may have worsened the prognosis.

Early-stage patients may be asymptomatic, and the lack of specific symptoms can make diagnosis difficult until fairly late in the clinical course. A variety of nonspecific symptoms and signs appear following the dissemination that occurs when the primary lesion ruptures and tumor cells are released into the abdominal cavity. The continuing accumulation of mucinous ascites occasionally causes partial or complete obstruction of the intestine [3]. PMP most often derives from lesions in the appendix followed by lesions in the ovary [33]. PMP rarely has lymphatic or hematogenous spread, but lung metastasis occurred in this patient a month after chemotherapy that was started following surgery. Ovarian metastasis occurred in this patient and previously in a patient with PMP associated with rectal mucinous adenocarcinoma [14]. Ovarian metastasis was suspected in a patient with PMP caused by sigmoid colon cancer but was not confirmed because surgery was not performed [15]. The clinical course of these three patients suggests that nonappendiceal mucinous adenocarcinoma predisposes to ovarian metastasis. Ovarian endocrine function and abundant lymphatic drainage and vasculature may favor the growth of metastatic tumors in such patients. The prognosis of PMCA is poor, and it may tend to metastasize to the ovary and other organs. Although CRS and HIPEC are recommended treatments, multitarget combination therapy appears to be a promising approach for PMP from CRC with BRAF V600 mutation.

## 4. Conclusions

This is the first systematically illustrated case of a PMP originating from a transverse colonic mucinous adenocarcinoma with ovarian metastasis. Lack of specific symptoms and signs delayed the diagnosis of PMCA until performing CRS. The characterization of this rare entity guides pathologists in this difficult diagnosis, especially regarding the importance of genetic testing for further potential treatment.

## Figures and Tables

**Table 1:** Reports of PMP caused by colorectal adenocarcinoma.

	Transverse colon cancer (this patient)	Rectal cancer [14]	Sigmoid colon cancer [15]
Age (years)	46	34	58
Gender	Female	Female	Female
Origin	Transverse colon	Rectum	Sigmoid colon
Metastasis	Ovary, lung	Ovary	Ovary was suspected
Tumor marker	CEA, 41 ng/mL	CEA, 364 ng/mL	CEA, 330.2 ng/mL
	CA19-9, 96 U/mL	CA19-9, 10 U/mL	CA19-9, 354.4 U/mL
	CA125, 240 U/mL	CA125, 30 U/mL	CA125, 166.7 U/mL
Treatment	CRS+chemotherapy	CRS+chemotherapy	Chemotherapy
Survival	29 months	<36 months estimated	<36 months estimated

## Data Availability

All the data supporting our findings are contained within the manuscript.

## Abbreviations

ASCO: The American Society of Clinical Oncology
BRAF: v-raf murine sarcoma viral oncogene homolog B1
CA125: Carbohydrate antigen 125
CA19-9: Carbohydrate antigen 19-9
CRP: C-reactive protein
CRS: Cytoreductive surgery
DPAM: Disseminated peritoneal adenomucinosis
ERK: Extracellular regulated protein kinases
EGFR: Epidermal growth factor receptor
FDA: The United States Food and Drug Administration
HIPEC: Hyperthermic intraperitoneal chemotherapy
KRAS: Kirsten rat sarcoma viral oncogene
LAMN: Low-grade appendicular mucinous neoplasms
mCRC: Metastatic colorectal cancer
MAPK: Mitogen-activated protein kinase
NSCLC: Non-small-cell lung cancer
NCCN: National Comprehensive Cancer Network
NRAS: Neuroblastoma rat sarcoma viral oncogene
ORR: Objective response rate
PFS: Progression-free survival
PMCA: Peritoneal mucinous carcinomas
PMP: Pseudomyxoma peritonei
PI3K-AKT: Phosphatidylinositol 3 kinase-protein kinase B
TP53: Tumor protein P53
XELOX: Capecitabine plus oxaliplatin.
